# Changes of tumor microenvironment in non-small cell lung cancer after TKI treatments

**DOI:** 10.3389/fimmu.2023.1094764

**Published:** 2023-03-06

**Authors:** Shanshan Chen, Jingyi Tang, Fen Liu, Wei Li, Ting Yan, Dangang Shangguan, Nong Yang, Dehua Liao

**Affiliations:** ^1^ Department of Pharmacy, Hunan Cancer Hospital, the Affiliated Cancer Hospital of Xiangya School of Medicine, Central South University, Changsha, China; ^2^ Lung Cancer and Gastrointestinal Unit, Department of Medical Oncology, Hunan Cancer Hospital, the Affiliated Cancer Hospital of Xiangya School of Medicine, Central South University, Changsha, China

**Keywords:** non-small cell lung cancer, driver mutation, tyrosine kinase inhibitor, tumor microenvironment, ICIs - immune checkpoint inhibitors

## Abstract

Non-small cell lung cancer (NSCLC) is the most common lung cancer diagnosis, among which epidermal growth factor receptor (EGFR), Kirsten rat sarcoma (KRAS), and anaplastic lymphoma kinase (ALK) mutations are the common genetic drivers. Their relative tyrosine kinase inhibitors (TKIs) have shown a better response for oncogene-driven NSCLC than chemotherapy. However, the development of resistance is inevitable following the treatments, which need a new strategy urgently. Although immunotherapy, a hot topic for cancer therapy, has shown an excellent response for other cancers, few responses for oncogene-driven NSCLC have been presented from the existing evidence, including clinical studies. Recently, the tumor microenvironment (TME) is increasingly thought to be a key parameter for the efficacy of cancer treatment such as targeted therapy or immunotherapy, while evidence has also shown that the TME could be affected by multi-factors, such as TKIs. Here, we discuss changes in the TME in NSCLC after TKI treatments, especially for EGFR-TKIs, to offer information for a new therapy of oncogene-driven NSCLC.

## 1 Introduction

Lung cancer is a prominent public health issue and has an enormous burden on society with the most common cause of cancer-related death worldwide. Non-small cell lung cancer (NSCLC), as the most common subgroup, accounts for approximately 85% of all lung cancer diagnoses (1). Due to the discovery of driver oncogenes, including mutation or rearrangement in epidermal growth factor receptor (EGFR), the Kirsten rat sarcoma (KRAS), anaplastic lymphoma kinase (ALK), ROS proto-oncogene 1 (ROS1), and rearranged during transfection (RET), and following the development of molecular-targeting agents, a new era of precision medicine has been started for NSCLC (2). Unfortunately, resistance is inevitable after treatments with these targeted therapies, which limits further clinical benefits. The resistance mechanisms of tyrosine kinase inhibitors (TKIs) for NSCLC are complex, multifaceted, and even less understood. For example, acquired resistance mechanisms to EGFR-TKIs include on-target resistance and off-target resistance, the former of which may show a second-site mutation in the EGFR kinase domain, preventing its binding with EGFR-TKIs, and the latter of which may result from alterations of the downstream components of the EGFR pathway, activation of alternative signaling pathways that bypass the primary drug targets, including MET amplification, or transition to another cell lineage, e.g., epithelial-to-mesenchymal transition (EMT) and SCLC transformation (2). The resistance mechanisms of ALK-TKIs are similar to that of EGFR-TKIs including ALK-dependent and ALK-independent resistance. Secondary mutations or amplification in the ALK tyrosine kinase domain are ALK-dependent, while the activation of bypass signaling pathways, drug efflux pump, and lineage changes is ALK-independent (3).

Therefore, it is urgent to develop a novel strategy to overcome the acquired resistance. Immunotherapy targeting the immunologic interaction between tumor cells and immune cells is vital to be explored. Immune checkpoint inhibitors are just the cases, which can stop immune evasion and recover immune surveillance. The on-market immune checkpoint inhibitors including programmed cell death protein-1 (PD-1), programmed cell death ligand-1 (PD-L1), and cytotoxic T-lymphocyte-associated protein 4 (CTLA-4) have shown efficacy with durable responses in some NSCLC patients (4, 5). However, only a subgroup of patients demonstrated benefits from immunotherapy, which was reported to be only 17%–21% (6).

The tumor microenvironment (TME) is composed of different cellular components including tumor cells, immune cells, fibroblasts, extracellular matrix components, microvesicles, and various associated cytokines and chemokines (7). The TME has been recently found to be a crucial factor for the efficacy of cancer treatment like targeted therapy or immunotherapy. On the one hand, the TME may affect the response of the tumor to therapy such as TKIs and immunotherapy. For example, the presence of CD4+ and CD8+ T cells in the TME may predict a better efficacy for EGFR-TKIs (8). Meanwhile, the resistance of EGFR-TKIs was also affected by the TME, such as TME stresses and autophagy. In addition, the efficacy of immunotherapy has been consistently shown to be correlated with the TME (9, 10). Close links have been shown between PD-L1 protein expression, the presence of tumor-infiltrating T cells (TILs), cytokines in the TME, and the immune response (11). For instance, both high infiltration of PD-1+ CD8+ T effector cells and PD-L1+ CD25+ CD4 TILs in the TME showed more effectiveness for PD-1/PD-L1 blockade immunotherapy (12), while a poor clinical efficacy of PD-1 inhibitors was observed in EGFR+ or ALK+ patients who possessed a non-inflamed TME or a so-called cold tumor, i.e., a paucity of TILs and an influx of immunosuppressive cells (13, 14). On the other hand, TKIs may potentially affect the TME, which in turn may have a great influence on the choice of treatments afterward. In clinical trials, even tumors without EGFR mutation responded well to EGFR-TKIs, which strongly suggested not only the tumor cells themselves but also potentially tumor-specific immune responses might be the targets of EGFR-TKIs. Except for inhibiting tumor cell viability directly, EGFR-TKIs could also enhance the immune function of T cells by downregulating the expression of PD-L1 and enhancing the production of interferon-γ (an indicator of T-cell function) as revealed in a cell study (15). However, an opposite relationship was also present. The immune response mediated by T cells was impaired by erlotinib through downregulating the c-Raf/ERK cascade and Akt signaling pathway. In addition, the pro-inflammatory cytokines such as interleukin (IL)-2 and IFN-γ were inhibited (16).

In spite of the positive effect of EGFR-TKIs on the TME, a respected synergistic antitumor effect with a combination of EGFR-TKIs and immunotherapy has not been presented in cell lines (15) concurrent with an EGFR mutation and PD-L1 upregulation, nor in patients (17, 18). Hence, it is necessary to know the changes of the TME in NSCLC after treatment with EGFR-TKIs or other TKIs to guide in choosing the best regimen. In this review, we endeavor to describe the TME in NSCLC before and after treatment with TKIs from the following three perspectives: the tumor-infiltrating immune cells; immune checkpoints expressed by tumor and immune cells, such as PD-L1; and cytokines or chemokines. As compared with their relative wild types, we will draw a baseline of the TME in different oncogene driver mutations NSCLC like EGFR, KRAS, and ALK. Changes in the TME in different mutation types after treatments with their relative TKIs will be depicted afterward. Furthermore, we will pay more attention to the influence caused by EGFR-TKIs, which are the most common driver mutations of NSCLC, accounting for 19%–67% varying with regions. Meanwhile, studies about the TME affected by TKIs have been mainly focused on EGFR-TKIs until now.

## 2 TME in oncogenic driver mutations

**Figure 1:**
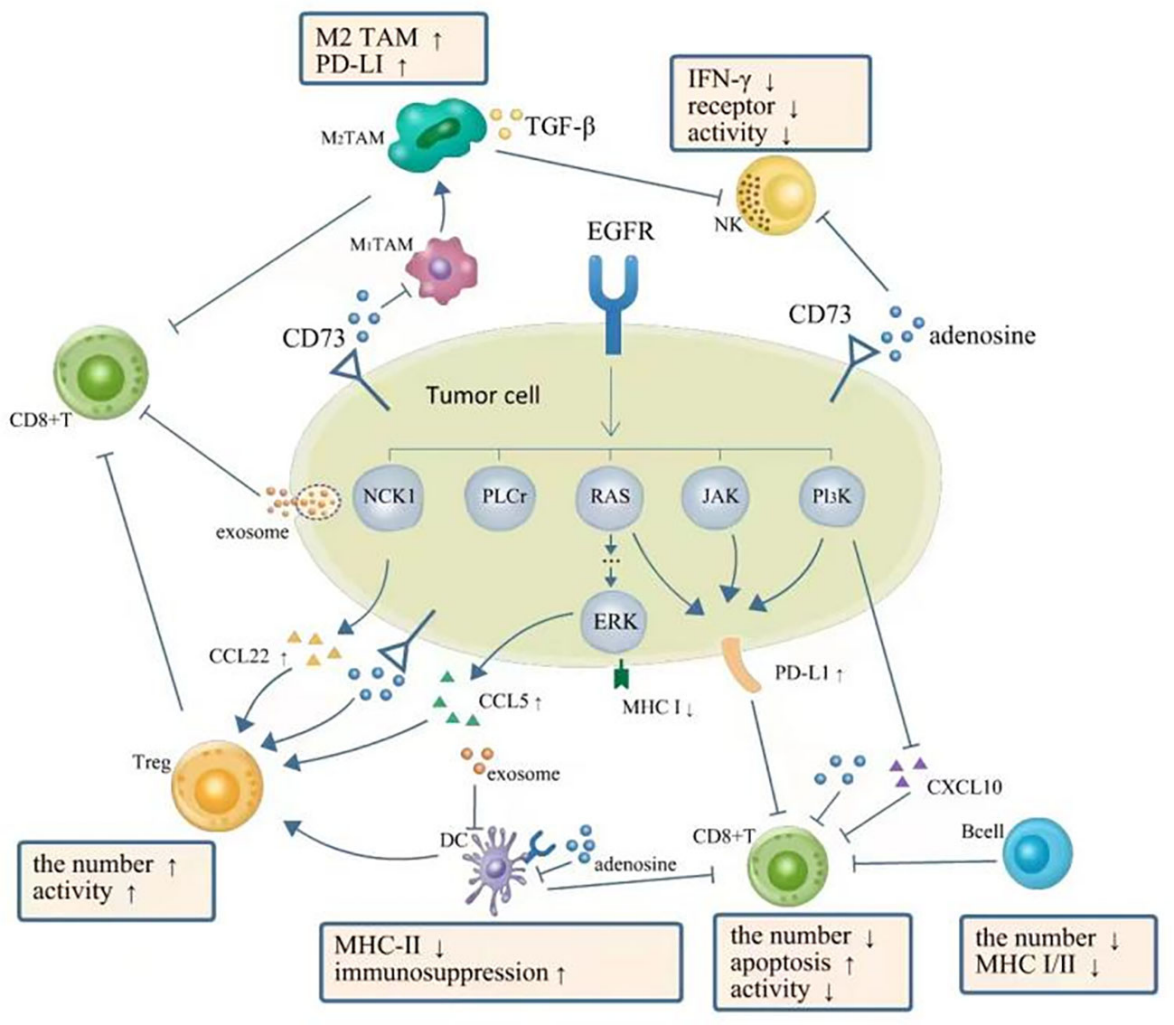
TME in NSCLC with EGFR mutation is presented as a phenotype of immunosuppression. EGFR signal upregulates expression of PD-L1 *via* PI3K-AKT, RAS-RAF-MEK-ERK, JAK-STAT3, and PI3K/AKT pathways, which further inhibit CD8+ T cells. The downregulation of CXC-chemokine ligand 10 (CXCL10) also recruits less CD8+ T cells. EGFR signal upregulates (CXCL22) and CC-chemokine ligand 5 (CCL5) resulting in the increase of Tregs. Adenosine as the immune-suppressive factor could be observed to be overproduced in EGFR-mutated cancer cells, which can directly suppress the process of NK cell killing, DC maturation, the function of macrophages, and the function and proliferation of cytotoxic T cells. A complex interplay also exists between different components of TME: DCs could impair the proliferation of CD8+ T cells. Tregs can actively attenuate and subvert the antitumor immune responses of CD8+ T cells by secreting cytokines such as TGF-β, IL-10, and IL-35. A high expression level of PD-L1 on M2 TAMs restricts the antitumor activity of CD8+ T cells. TGF-β secreted by M2 TAMs can also directly suppress the process of NK cell killing. Moreover, exosomes can repress the function of DCs and the proliferation of CD8+ T cells. TME, tumor microenvironment; NSCLC, non-small cell lung cancer; EGFR, epidermal growth factor receptor; NK, natural killer; DC, dendritic cell; TAM, tumor-associated macrophage.

As a heterogeneous disease, NSCLC may have multiple histologic subtypes that harbor disparate mutational profiles such as EGFR, ALK, and KRAS mutations, which are the known predominant genetic drivers (19). The TME may be modified by different driver mutations, which could be revealed as differences in tumor-infiltrating immune cells, immunomodulatory molecules, cytokines, chemokines, etc. (20). For instance, an immunosuppressive TME with inactive TILs was shown in EGFR mutant NSCLCs compared with the wild ones, which was inferred from a low expression of Ki67 (markers of proliferation) and granzyme B (markers of cytotoxicity) of T cells. The situation was reversed completely for KRAS-mutant ones, as Ki67 and granzyme B were highly expressed by TILs compared with their wild types (21). As so, we firstly review the baseline of TME in different oncogenic driver mutations compared with their relative wild types (**Table 1**) especially for the EGFR-MT types and the wild types (**Figure 1**).

**Table 1:** Components of TME in mutuation type compared with the wild type.

Mutuation type	Components in TME	Components of TME	Mutuation type Compared with the Wild type	Immune activity
EGFR	Immune cells	CD8+T cells	↓	↓
		TFHcells	↓	↓
		Treg	↑	↓
		B cells	↓	↓
		NK cells	activity↓	↓
		M2-TAMs	↑	↓
		Immunesuppresive DC	↑	↓
		DCs	activity↓	↓
	immunemodulatory molecules	MHC	↓	↓
		PD-L1	↑ or↓	↓or↑
		CD73	↑	↓
		CD47	↑	↓
		ILT4	↑	↓
	Cytokines and chemokines	CCL18, CXCL1 and CXCL3	↑	↓
		CCL5 and CCL22	↑	↓
		CXCL10,CXCL13	↓	↓
ALK	Immune cells	CD4+T(activating immune milieu)	↓	↓
	Immune checkpoints	PD-L1	↑	↓
KRAS	Immune cells	CD8+T cells	↑	↑
MET	Immune cells	CD8+T cells	↑	↑

The symbol of “↑” means the content or the acticity of the relative ingredients in TME have been up-regulated, while the symbol of “↓” means down-regulation of the content or the acticity of the relative ingredients in TME.

### 2.1 Tumor-infiltrating immune cells

#### 2.1.1 CD8+ T cells

As the major players in immunotherapy today, cytotoxic CD8+ T cells (CD8+ T cells) undertake cytolytic activities against tumor cells *via* the following steps: firstly, effector CD8+ T cells are activated upon the recognition of an antigen major histocompatibility complex (MHC) on the tumor cell; then, granules that contain perforin, granzyme, and the Fas ligand are released by CD8+ T cells upon successful recognition to give full rein to effector functions.

Pre-clinical and clinical evidence has shown that the number of CD8+ T cells (22–25) could be reduced and the apoptosis of T cells could be induced by EGFR signaling through PD-L1 upregulation (15). Meanwhile, exosomes secreted by EGFR-mutant NSCLC cell lines were also capable of promoting CD8+ T-cell apoptosis (23). In addition, the activity of CD8+ T cells was reduced in EGFR-mutated NSCLC with lower fractions of Gzmb+ CD8+ T cells (22) or CD8+ Ki67+ T cells (24). This result was verified by a single-cell transcriptome analysis that a significantly lower expression of marker genes of active CD8+ T cells was found in the EGFR-mutation group than in the wild-type group (26).

CD8+ tissue-resident memory T cells (CD8+ TRM cells) residing in tumor tissues were found to be able to protect against cancer by secreting cytokines to prompt tumor-immune equilibrium and/or *via* CD103-enhanced tumor cell killing (27). Traditionally, CD8+ TRM cells have been identified by a marker of CD103 expressed on the surface. These CD8+ TRM cells were proved to be associated with improved disease outcomes and patient survival. For instance, among NSCLC patients with a similar degree of infiltration by CD8+ T cells, patients whose tumors exhibited high CD103 expression fared better than those with low CD103 expression (28). Compared to EGFR-WT, the abundance of CD8+ TRM cells was much lower in EGFR-MT (26, 29). In addition, the expression of granzyme B (GZMB) and genes involved in CD8+ TRM cell activation has been shown to be lower with EGFR-MT than the wild type, indicating that CD8+ TRM cells in EGFR-MT tumors have a lower anti-neoplastic activity to kill cancer cells (29). The persistence of the CD8+ TRM cell state in the TME may have a critical effect on killing tumor cells effectively since CD8+ TRM cells can secrete a majority of cytolytic enzymes such as perforin and GZMB. As proved in EGFR-MT lung tumors, the persistence of the CD8+ TRM cell state is impaired (29).

Furthermore, CD8+ T cells may vary with different mutation sites. The infiltration of CD8+ T cells was significantly higher in EGFR L858R samples than in EGFR exon 19 deletion samples. CD8+ T cells were enriched in KRAS-mutant NSCLC compared with the wild type (30) and even EGFR-MT (31). MET-mutated patients also had more CD8+ T-cell infiltration than MET wild type (32).

#### 2.1.2 CD4+ T cells

CD4+ T cells engaged in adaptive immunity are another major immune cell in the TME in addition to CD8+ T cells. Various subtypes of CD4+ T cells have different and even opposite functions like antitumor activities or tumor-promoting processes. Helper T cells (TFH) belong to the former, which can activate other immune cells, including cytotoxic T cells and B cells. It is reported that the maturation of antibody affinity was promoted by TFH (33), and the production of CXCL13, a major chemoattractant in driving B cells to follicles, was also promoted (34). As immunosuppressive CD4+ T cells, regulatory T cells (Treg cells) expressing forkhead box protein P3 (FOXP3) (35) play key roles in suppressing antitumor immunity in the TME (36) through the EGFR/GSK-3/Foxp3 pathway *in vitro* and *in vivo* (37). Tregs can attenuate and subvert the antitumor immune responses of CD4+ T cells, CD8+ T cells, and natural killer (NK) cells by secreting TGF-β, IL-10, and IL-35. In a single-cell transcriptome analysis of TILs, the proportion of CXCL13-producing TFH-like cells was discovered to decrease, whereas the proportion of CD4+ T cells contributing to immunosuppression increased in EGFR-MT tumors (29). Tregs were highly detected in EGFR-MT lung adenocarcinoma (LUAD) samples, which was verified by transfected cell line-derived tumors (22). In another study, the abundance of Tregs was decreased in EGFR-MT, while compensated by expressing a significantly high level of genes, which are positively associated with the activity of Treg (TSC22D3 encoding GILZ43 and NR4A244) (29).

Other oncogenic driver mutations may display some differences for CD4+ T cells. Resting memory CD4+ T cells were enriched and activated memory CD4+ T cells were lacking in ALK rearrangements, yielding an uninflamed phenotype with immune ignorance (38). A higher CD4+ T-cell infiltration resulting in activating the immune milieu was detected in MET-mutated patients than in MET wild-type ones (32).

#### 2.1.3 B cells

Intratumoral B lymphocytes are an integral part of the lung tumor microenvironment. B cells in the TME, in a recent study, showed a positive effect on the response of immunotherapy and survival in cancer patients by forming tertiary lymphoid structures (TLSs), where the presentation of a tumor-derived neoantigen to T cells by B cells occurred and then activation of T cells followed. B cells were significantly decreased in EGFR-MT compared to EGFR-WT by single-cell RNA sequencing (scRNA-seq). In addition, the ability of B cells to present antigen to both CD8+ and CD4+ T cells was impaired in EGFR-MT than EGFR-WT as a result of the lower expression of MHC class I and II, respectively (29).

#### 2.1.4 NK cells

NK cells can build a coordinated antitumor immune response with their cytotoxic effector functions and their capacity to interact with other immune cells. NK and NKT cells can kill cancer cells directly *via* cytotoxic molecules, such as IFN-γ secretion, independent of antigen specificity. Not only was the IFN-γ secretion from the NK cells inhibited by NSCLC cell lines H1975 (EGFR L858R+T790M), but the surface ligands interacting with receptors on NK cells (ULBP1, ULBP2, and MICA) were also downregulated after being co-cultured with H1975 cell line (39). In contrast, cytotoxic NK cells expressing more genes involved in activation and cytotoxicity were decreased, whereas NKT cells with relatively low cytotoxicity were increased in EGFR-MT tumors, which was revealed by single-cell transcriptome analysis (29). A gene set enrichment analysis based on The Cancer Genome Atlas (TCGA) database also verified this to some degree where more NKT cells existed in EGFR-MT patients, while three pathways upregulating the activity of NK cells were downregulated (40).

#### 2.1.5 TAMs

Tumor-associated macrophages (TAMs) are classified as the M1 macrophages and the M2 macrophages. M1 TAM tends to enhance the immune response in the TME by producing chemokines and cytokines to recruit the cytotoxic CD8+ T and NK cells to destroy the tumor cells (41). However, M2 TAMs can protect the cancer cells from antitumor immune responses and promote their proliferation, angiogenesis, and metastasis during tumor progression. M2 TAMs were associated with high levels of PD-L1 expressed on both tumor cells and tumor-infiltrating immune cells in patients, which restrict the antitumor activity of T cells as a result (42). TGF-β secreted by M2 TAMs could also impede the cytotoxicity of NK cells (43). Oncogenic EGFR signaling greatly resulted in the expansion of alveolar macrophages (AMs; lung cancer tissue-resident macrophages) consistent with an M2-macrophage phenotype harboring reduced expression of costimulatory molecules, such as CD40, CD80, and CD86, as well as downregulated MHC-II expression (44). Similarly, an enhanced M2-like polarization and migration ability of TAMs in EGFR-MT NSCLC cells were found to be promoted by immunoglobulin-like transcript 4 (ILT4; a key immunosuppressive molecule), which was upregulated *via* EGFR-AKT/-ERK1/2 signaling (45). Moreover, M2 TAMs were found less in the tumor stroma of EGFR-MT NSCLC compared with EGFR-WT in one study (46).

#### 2.1.6 DCs

Dendritic cells (DCs), with the antigen-presenting ability, are capable of modifying the TME and promoting the antitumor immune response *via* the activation of antigen-specific CD8+ T cells (47). Higher proportions of CD1A+DC and CD1C+DC were detected in the EGFR-MT group, which presented to be more immune-suppressive with higher expression of immunosuppressive chemoattractants, i.e., CCL4, CCL17, CCL22, CXCL2, and CXCL17 (26). In detail, CCL17 and CCL22 as ligands of CCR4 are involved in the increase of Tregs in tumors (48). CCL4, CXCL2, and CXCL17 are chemoattractants of immune-suppressive cells such as myeloid-derived suppressor cells (MDSCs), Tregs, and macrophages (49–51). The expression of MHC-II indicated the maturation of DCs in tumors. There was almost no difference in the density of total DCs between the EGFR-19del tumor-bearing mice and the EGFR-WT ones, but DCs from the EGFR-19del group had a lower expression level of MHC-II and produced much less IL-12p40, which could enhance the downstream cytotoxic immune response, indicating that less activity of DC was shown in the EGFR-19del tumor (24). Moreover, the activity of DCs was impeded by EGFR-19del LLC cells *via* the uptake of the exosomes, derived from their own (24). Furthermore, the proliferation of CD8+ T cells could also be impeded by DCs in EGFR-19del tumors (24).

### 2.2 Immunomodulatory molecules

#### 2.2.1 MHC I and MHC II

MHC can present tumor antigens to T cells, including two kinds of MHC. MHC class I molecules (MHC I), expressed on the surface of almost all nucleated cells, present antigenic information to CD8+ T cells, while MHC class II molecules (MHC II), expressed on the surface of antigen-presenting cells, such as B cells, macrophages, and DCs, present antigenic information to CD4+ T cells. EGFR activation downregulated the expression of MHC I and MHC II by repressing CIITA, an MHC II transactivator, which decreased the expression of MHC I and MHC II (52). Furthermore, it was the MEK-ERK pathway but not the PI3K-AKT pathway that mediated the downregulation of MHC class I expression in EGFR-mutated cell lines (53).

#### 2.2.2 PD-L1

PD-L1, one of the critical immune checkpoint molecules, is modulated *via* two different mechanisms in NSCLC: drive genetic alterations and inflammation. Intrinsically, PD-L1 expression is upregulated by aberrant oncogenic EGFR and ALK signaling. Despite an inexact oncogenic induction mechanism, it has been suggested that the PI3K-AKT, RAS-RAF-MEK-ERK, and JAK-STAT3 pathways may be involved in the induction of PD-L1 (15) (54–56). In addition, PD-L1 expression has been found to be regulated by IFN-γ *via* IRF1 signaling, JAK/STAT3, and PI3K/AKT (22, 57).

The correlation between the EGFR signaling pathway and PD-L1 expression remains largely controversial. Most pre-clinical data displayed upregulation of PD-L1 in EGFR-mutated NSCLC cell lines (15, 22, 58, 59) by detecting either the protein or mRNA level of PD-L1. However, the results revealed by immunohistochemistry (IHC) did not turn out that way. Lung cancer cell lines with EGFR-MT have a low expression of PD-L1 (60). Moreover, the expression of PD-L1 detected by IHC or mRNA was significantly lower in EGFR-MT NSCLC than in the wild types as revealed in a large number of clinical trials (25, 38, 61). A pooled analysis of 15 public studies also demonstrated that PD-L1 expression in EGFR-mutation patients was decreased, which was confirmed by analysis of the protein and mRNA profiles of PD-L1 in the TCGA and Guangdong Lung Cancer Institute (GLCI) (62).

Other mutant types seemed to have a higher PD-L1 expression than EGFR-MT. For example, a significantly higher expression of PD-L1 mRNA was detected in ALK-positive tumors compared to EGFR-positive ones (25). In addition, the expression of PD-L1 detected by IHC was higher in KRAS-mutant NSCLC patients compared with KRAS wild-type, EGFR-MT, and ALK-MT patients (38). A higher PD-L1 expression in MET-MT NSCLC patients detected by IHC was reported than in EGFR-MT and KRAS MT subgroups (63).

#### 2.2.3 CD73

CD73 is a novel negative immunomodulatory protein that is widely expressed in immune cells and cancer cells. It contributes to an immunosuppressive TME by converting ATP to adenosine, which can impair the activity of multiple immune cells including T cells, macrophages, NK cells, and DCs (64), and enhance the suppressive capacity of Tregs and MDSCs (65, 66). CD73 was found high in EGFR-MT NSCLC tumor cells (67, 68), which may be a novel immune-relevant drug target to inhibit the growth of tumors. For example, a CD73-targeted antibody–drug conjugate could remodel an immunosuppressive TME in multifaceted ways like diminishing levels of TAMs, MDSCs, and tumor vasculature, which inhibited the growth of tumors in KRAS-mutant NSCLC mice (69).

#### 2.2.4 CD47

CD47 is a phagocytic checkpoint protein over-expressed on many tumor cells, which inhibits phagocytosis of the macrophages *via* binding to signal regulatory protein α (SIRPα) expressed on macrophages (70). CD47 mRNA was expressed more highly in patients with EGFR-MT than in patients with KRAS mutations, ALK fusion, or wild types. Moreover, a significant increase in CD47 expression was discovered in the gefitinib-resistant cell surface relative to the parental cell lines (71).

#### 2.2.5 ILT4

ILT4 is an immunosuppressive receptor mainly expressed in MDSCs including monocytic MDSCs (mMDSCs) and granulocytic MDSCs in the TME (72). ILT4 can mediate immunosuppression by negatively regulating the antigen presentation of DCs, phagocytosis of neutrophils, and the maturation of macrophages. A more pro-inflammatory state could be induced by inhibiting ILT4 so that more M1 phenotypes shift from the M2 phenotype (73). ILT4 was upregulated in NSCLC with EGFR activation by EGFR-AKT/-ERK1/2 but not NF-κB signaling, which further blocked the infiltration and cytotoxicity of T cells (45).

#### 2.2.6 Others

Many other suppressive immune checkpoints, such as PD-1, T-cell immunoglobulin and mucin domain-containing protein 3 (TIM3), and lymphocyte activation gene 3 (LAG3), were highly expressed by TRM cells (74, 75), while CD8+ T cells expressing PD-1, TIM3, and LAG3 were lower in the EGFR-MT group, indicating less CD8+ TRM and a more immunosuppressive TME (26).

### 2.3 Cytokines and chemokines

Cytokines and chemokines produced by tumor cells or non-tumor cells are also of great significance to the TME, which can be categorized as either immune-suppressive factors or immune-promoting ones. Adenosine, as the immune-suppressive factor, was overproduced in EGFR-MT cancer cells (76), which can directly suppress the process of NK cells killing, the maturation of DCs, and the function and proliferation of cytotoxic T cells, although the detailed mechanism(s) remains undetermined. IL-1β, another immune-suppressive cytokine to recruit MDSCs toward tumor sites, was observed to be strongly expressed in EGFR-positive-specific cells (77). Some immunosuppressive chemokines, such as CCL18, CXCL1, and CXCL3, were more highly expressed in the EGFR-MT group than the wild-type group (26), with CCL18 recruiting Tregs (78), CXCL1 recruiting TAMs (79), and CXCL3 inducing MDSCs (80). Both CCL5 and CCL22 were associated with the recruitment of Tregs, which may induce the exhaustion of CD8+ T cells. A prominent temporal increase of CCL5 was also demonstrated in EGFR-MT through increased MAPK/ERK signaling (81). The CCL22 was also elevated in EGFR-MT *via* JNK/c-Jun activation (22).

In contrast, immune-promoting factors were decreased in EGFR-MT tumors. For instance, CXC-chemokine ligand 10 (CXCL10), which could reportedly recruit CD8+ T cells (82), was downregulated (22) *via* the PI3K-AKT pathway in EGFR activation. Concurrently, IRF1 as a positive regulator of CXCL10 was also decreased (22). The expression of CXCL13 secreted by T cells, which could mediate the recruitment of B cells to tumors, was lower in the EGFR-positive group. As a result, these cytokines and chemokines may model an immunosuppressive TME, especially in EGFR-MT NSCLC.

## 3 TME after TKI treatments

**Figure 2:**
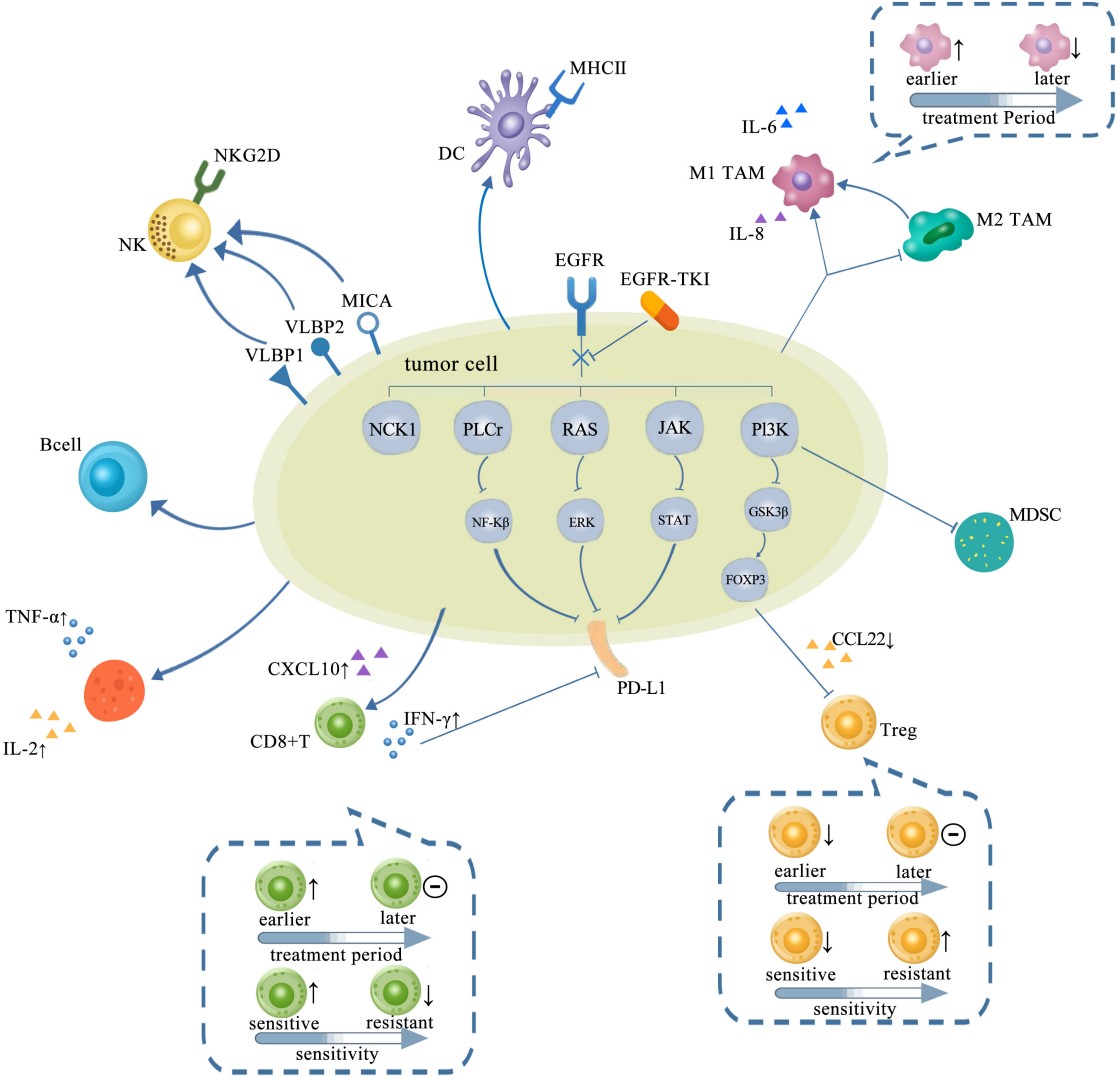
TME in NSCLC with EGFR mutation changed with treatments of TKIs, which is presented as more immunostimulatory. EGFR-TKIs may decrease the expression of PD-L1 *via* blocking NF-κB, ERK, and STAT. Generally, a higher CD8+ T-cell infiltration in the TME after TKI treatment is concurrent with a higher CXCL10 expression. *CD8+ T cells changed with treatment period and sensitivity:* earlier, the number of CD8+ T cells elevated and later remained unchanged. Also, the infiltration of CD8+ T cells elevated upon sensitive TKI treatments while decreased upon resistance. The infiltration and function of Tregs were significantly reduced by inhibiting the GSK-3β pathway concurrent with downregulated CCL22. *Tregs also changed with treatment period and sensitivity:* earlier, the number of Tregs decreased and later remained unchanged. The number of Tregs decreased upon sensitive TKI treatments while elevated upon resistance. Upregulation of B cells is detected after TKI treatment. CD4+ T cells involved in the antitumor activity are mostly increased by TKIs with increasing TNF-α and IL-2. The interaction between NK cells and human lung cancer cells was enhanced by TKI *via* upregulation of NKG2D on NKs and NKG2D ligands, such as ULBP1, ULBP2, and MICA on tumor cells. A high degree of DC infiltration concurrent with a higher MHC class II expression on their surfaces was obtained after TKIs. The abundance of M1-TAMs is usually upregulated by TKIs at earlier but downregulated later. However, M2-TAMs usually tend to decrease and even shift to M1-TAMs by TKIs. TME, tumor microenvironment; NSCLC, non-small cell lung cancer; EGFR TK2, epidermal growth factor receptor; TKIs, tyrosine kinase inhibitors; DC, dendritic cell; TAM, tumor-associated macrophage.

TKIs may remodel the TME by regulating the components such as tumor-infiltrating immune cells, immunemodulatory molecules, cytokine and chemokines, etc (**Table 2**). These components might even be varied with space and time after TKI treatments. As we know, TME performed as a significant space in targeted therapy and immunotherapy. Hence, it is of a vital importance to comprehensively reveal and understand the change of TME after TKI treatments. The effects caused by EGFR-TKIs has been depicted in **Figure 2**.

**Table 2:** Changes of TME after treatments with TKIs.

Mutuation type	Components in TME	Components of TME	Changes after TKIs	Immune activity
EGFR	Immune cells	CD8+T	↑^*,#,$^	↑^*,#,$^
		Effector CD4+ T cells	↑	↑
		Tregs	↓^*,#,$^	↑^*,#,$^
		B cells	↑or -	↑or -
		M1-TAMs	↑	↑
		M2-TAMs	↓^#^	↑
		NK cells	↑^#^	↑
		MDSCs	↓or suppressive activity ↓	↑
	immunemodulatory molecules	**MHC**	↑	↑
		PD-L1	↓or↑^#^	↓or↑^#^
		CD47	↓	↑
		ILT4	↓	↑
	cytokines and chemokines	TNF-α, IFN-γ, IL-2	↑	↑
		IL-6	↑	↑
		IL-8	↑	↑
		TNF	↑	↑
		CXCL10	↑	↑
		CCL22	↓	↑
		CCL2, CCL5	↑	↓
		CCL3, CXCL1	↓	
		CXCR4	↑	
ALK	Immune cells	CD8+T	activity↓	↓
		NK cells	↑	↑
	immunemodulatory molecules	MHC	↑	↑
		PD-L1	↑#	

* The change was TKI treatment period dependent.

^#^ The change was TKI sensitivity dependent.

$ The change was TKI treatment regimens dependent.

↑ means up-regulation of the content or the acticity of the relative ingredients in TME.↓ means down-regulation of the content or the acticity of the relative ingredients in TME.

### 3.1 Tumor-infiltrating immune cells

#### 3.1.2 CD8+ T cells

Generally, we found a higher CD8+ T-cell infiltration in the TME after TKI treatment (22, 53, 83, 84), concurrent with higher CCL2, CXCL10, and CCL5 expression than before (22, 85). Studies also revealed that IFN-γ produced by CD8+ T cells was also increased after 2 weeks of erlotinib treatment (85). In flow cytometry, not only the infiltration of CD8 cells was affected by osimertinib treatment, but also the activity was less exhausted revealed by a lower expression of TIM3+ and PD-1+ CD69− (86). Moreover, according to their migration and adhesion abilities marked as CD62L and CD44 expression, respectively, the tumor-infiltrating CD8+ T cells could be further classified as naïve or effector CD8+ T cells (87, 88). Naïve CD8+ T cells characterized by high CD62L and low CD44 were not affected by erlotinib. On the contrary, the percentage of effector CD8+ T cells, characterized by low CD62L and high CD44, was significantly increased afterward (85). Despite that, an increase appeared in the densities of effector CD8+ TILs and granzyme B expressed by CD8+ T cells showed no significant difference after erlotinib treatment in lung tumor tissue, implying that CD8+ T cells in the TME did not degranulate (85). Unexpectedly, the effect of erlotinib on CD8+ T cells was mediated by tumor regression indirectly, instead of the direct influence of erlotinib, which was verified by the following evidence. On the one hand, both the EGFRL858R+T790M mutant mouse model and healthy non-tumor-bearing mouse model remained unresponsive to erlotinib, indicating that there is still a low number of and functionally impaired CD8+ lymphocytes even after TKI treatment. On the other hand, the increase of CD8+ lymphocytes after erlotinib treatment was not a result of the increased proliferation, which could be inferred from their decreased Ki-67 positivity (85).

The change of CD8+ T cell may vary with treatment period, TKI resistance, and even treatment regimens. *Treatment period dependent:* in EGFR-MT lung tumors, CD8+ T cells were confirmed to be elevated only on days 1 and 2, and no significant difference existed from day 3 to day 7 during the sensitive EGFR-TKI treatment (89). In the early period, afatinib inhibited the proliferation of CD8+ T cells in NSCLC patients by targeting CAD, which is critical for *de novo* pyrimidine biosynthesis. After long-term treatment, the proliferation of CD8+ T cells rebounded unexpectedly, suggesting that afatinib may only have a temporary immunomodulatory effect on CD8+ T cells (90). *TKI resistance dependent:* infiltration of CD8+ T cells in the TME was significantly increased after the sensitive TKI therapy as shown in the above-mentioned studies, while CD8+ T cells appeared significantly decreased upon EGFR-TKI resistance as demonstrated in 21 cases of EGFR-MT NSCLC (91). A similar phenomenon was revealed in biopsies from EGFR-MT NSCLC patients by single-cell RNA sequencing, suggesting that more infiltration of CD8+ T cells was associated with an effective EGFR-TKI treatment while less with a resistant EGFR-TKI treatment (92). *TKI treatment regimen dependent:* the antitumor effects even immunoregulation of TKIs may vary with their treatment regimens. For instance, a hypofractionated EGFR-TKI treatment (hypoTKI: a treatment with a high dose but low frequency), rather than a hyperfractionated EGFR-TKI treatment (hyperTKI: a treatment with a low dose and daily), appeared to be more effective in preventing tumor relapse in Her2/EGFR-driven tumors. Markedly increased CD8+ T cells were observed in the TME at 72 h after hypoTKI but not hyperTKI. Moreover, hypoTKI could enhance the production of interferon (IFN) and CXCL10 *via* the Myd88 signaling pathway, which could further enhance the infiltration and reactivation of tumor-specific T cells (93). However, these results need to be verified further in EGFR-MT NSCLC.

CD8+ T cells in the TME might also be affected by other TKIs like ALK-TKIs. In ceritinib-resistant tumors, CD8+ T cells lack cytotoxicity and tumor antigen specificity, meanwhile surrounded by PD-L1-expressing cells and Treg cells, implying that CD8+ T cells in ceritinib resistance may just be bystanders (94).

#### 3.1.3 CD4+ T cells

CD4+ T cells involved in antitumor activity are mostly increased by TKIs. Effector CD4+ T cells, which are potent to enter sites of inflamed peripheral tissues, were significantly increased after erlotinib treatment instead of naïve ones, which were residing in the lymph nodes (85). Furthermore, CD4+ T cells converted to an activated phenotype producing increased cytokines like IL-2 and TNF-α after erlotinib treatment (85).

However, CD4+ T cells are involved in tumor promotion or immunosuppression; e.g., Tregs are usually decreased by TKIs. As AREG/EGFR signaling can enhance Foxp3 expression by inhibiting the GSK-3β/β-TrCP pathway, the Treg infiltration and its function were significantly reduced after erlotinib (22) or gefitinib (53) treatment by the inhibition of Foxp3 expression. A limited reduction of Tregs and a significant decrease of Tregs/CD8+ T-cell ratio were observed after erlotinib treatment, suggesting a shift toward a more immune-active TME (85).

Just like CD8+ T cells, the changes of CD4+ T cells in the TME were also affected by the TKI treatment period, TKI sensitivity, and TKI treatment regimens. *TKI treatment period dependent:* in EGFRL858R-driven and EGFR19DEL/T790M-driven tumors, the infiltration of Tregs reduced only on the first 2 days, similar on day 3 and day 7 in the responsive-treated group (89). *TKI sensitivity dependent:* Foxp3+ Treg cells showed a significant increase after gefitinib resistance (94). Ceritinib as an ALK-TKI also increased the number of Foxp3+ Treg cells after resistance, concurrent with upregulated genes associated with Treg differentiation and function (95). *TKI treatment regimens dependent:* in Her2/EGFR-driven tumors, hypoTKI, not hyperTKI, markedly increased CD4+ T cells in the TME after treatment, which needs to be expanded in EGFR-MT lung cancer (93).

#### 3.1.4 B cells

B cells have been usually upregulated after TKI treatments demonstrated by increased infiltration of B cells in EGFR-MT samples by single-sample gene set enrichment analysis (ssGSEA) (83). However, some differences appeared in other investigations. For instance, the B-cell population showed no significant changes in either EGFR L858R-driven or T790M-driven tumors within 24 h after their sensitive TKI therapy (84). Even TKI treatment regimens make a difference to B cells in the TME. Unlike hyperTKI, hypoTKI markedly increased B cells in the TME after treatment (93).

#### 3.1.5 TAMs

The abundance of M1-TAMs is usually upregulated by TKIs. Upon erlotinib treatment, increased infiltration of macrophages concurrent with significantly higher expressed MHC class II on the surfaces were seen in EGFRL858R-driven tumors, implying that antigen-presenting capabilities increased (84). Furthermore, an early increase of M1-TAM was observed in both L858R-driven and EGFR19DEL/T790M-driven models with sensitive TKI treatment, shown by an elevation on days 1 and 2, but a reduction by day 7 (89). By contrast, M2-TAMs usually tend to decrease and even shift to M1-TAMs by TKIs. CD206+ macrophages (M2-like) reduced from day 1 to day 7 after sensitive TKI treatments in mouse models, suggesting EGFR-TKIs could inhibit M2-like polarization (89). Another study also revealed M2-AMs were decreased to baseline levels after 2 weeks of erlotinib delivery in an L858R-driven mouse model (44). M2-like AMs decreased significantly after erlotinib treatment in the lungs of tumor-bearing mice due to probably decreased proliferation, which could be inferred from a lower percentage of Ki-67+ positivity. Meanwhile, a switch to pro-inflammatory M1 macrophages after erlotinib treatment was supported by the increased expression of CD86 and Irf5 (85). Furthermore, a shift of TAMs toward M1-like by osimertinib was mediated *via* the cGAS/stimulator of interferon genes (STING) pathway (96). Hence, the change of TAMs might be promoted by the sensitivity of the tumor to TKIs, i.e., sensitive or resistant. Less and more macrophage infiltrations were observed, respectively, after sensitive and resistant EGFR-TKI treatments in biopsies from EGFR-MT NSCLC patients by a single-cell RNA sequencing analysis (92).

#### 3.1.6 NK cells

The cytotoxicity of NK cells could be enhanced by gefitinib through multiple mechanisms as revealed in EGFR L858R+T790M lung cancer. Firstly, gefitinib could enhance the interaction between NK cells and tumor cells *via* the upregulation of NKG2D on NKs and NKG2D ligands, such as ULBP1, ULBP2, and MICA on tumor cells (97). Secondly, the immunomodulation of gefitinib was proved to be mediated by stat3 inhibition, which could be phosphorylated by activated EGFR and exerts an inhibitory effect on antitumor NK cell immunity. Finally, gefitinib-induced autophagy was found able to accelerate mannose-6-phosphate receptor upregulation, by which way the NK cytotoxicity was enhanced (39). Changes in NKs in the TME may also be affected by the sensitivity of TKIs. The infiltration of NK cells increased significantly in EGFR L858R-driven lung tumors within 24 h after a sensitive erlotinib therapy, while no significant change was discovered in EGFRL858R/T790M-driven tumors after an unresponsive erlotinib treatment (84). A dramatic increase in the infiltration level of NK CD56dim cells in ALK-r samples was also observed after treatment with ALK-TKIs by a ssGSEA (83). NK cells became activated and more mature when treated by a combination of MEK inhibitors (a downstream signaling pathway of KRAS) and CDK4/6 inhibitors, with the expression of CD107a and other activating receptors and an increase in the expression of genes associated with NK cell maturation and cytotoxicity (98). This was also verified by the increase of genes linked to the recruitment, proliferation, and activation of NK cells (98).

#### 3.1.7 DCs

The infiltration of DCs has increased with a 24-h responsive erlotinib treatment in EGFR L858R-driven tumors. Meanwhile, DCs gained a higher antigen-presenting capability as a result of a higher MHC class II expression (84). Consistent with this, a significant increase of CD103+ dendritic cells was also observed in EGFR-MT mice after erlotinib treatment for 2 weeks (85).

#### 3.1.8 MDSCs

In addition to TAMs and Tregs, MDSCs are also a key immunosuppressive cell type that participated in tumor immune escape. Unlike other immune cells mentioned above, the decrease of MDSCs was independent of hypoTKI or hyperTKI treatment (93). In spite of an increase in infiltration of MDSCs shown in EGFR L858R-driven tumors upon erlotinib treatment, the suppressive activity of MDSCs might be impaired *via* decreased STAT3 phosphorylation, which needs further investigation (84). A significant increase of MDSCs took place in EGFR-MT mice during the entire sensitive EGFR-TKI treatment period was revealed in another study (89). With MDSC subtypes further evaluated, the mononuclear MDSCs were elevated throughout the treatment period, while polymorphonuclear MDSCs stayed unchanged, suggesting that a sensitive EGFR-TKI therapy could recruit MDSCs to tumor sites and change their phenotypes in EGFR-driven lung tumors (89).

### 3.2 Immunomodulatory molecules

#### 3.2.1 MHC

The expression of MHC I and MHC II is usually upregulated by EGFR-TKIs. For instance, an increased MHC-I expression at both mRNA and protein levels may be caused by sensitive EGFR-TKI treatments even in patients harboring a secondary T790M mutation. The precise mechanism may include the MEK-ERK pathway but not the PI3K pathway (53). Other TKIs like ALK inhibition proved to be able to upregulate HLA-I expression *via* ALK-MAPK signaling *in vitro* (99).

#### 3.2.2 PD-L1

The expression of PD-L1 is complex and even controversial after treatment with TKI. Inhibiting the activity of EGFR by sensitive EGFR-TKIs was likely to downregulate the expression of PD-L1 both *in vitro* and *in vivo* (89). This reduction was found to be associated with the NF-κB signaling pathway (15, 58, 100) and its downstream pathway including pAKT, pERK, and pSTAT3 (59). Osimertinib (55, 58, 101) could downregulate PD-L1 not only at the mRNA level but also at the protein level. For example, proteasomal degradation of PD-L1 protein was prompted by osimertinib *via* a novel PD-L1 E3 ligase (MARCH8) (102). However, an increased trend of PD-L1 was observed in other studies. Five of 13 (38%) EGFR-MT NSCLC patients showed major increases in PD-L1 expression after being treated with EGFR-TKI, implying that EGFR-TKI might tend to promote the expression of PD-L1 in EGFR-MT (103). Meanwhile, as revealed in a mouse model, the expression of Cd274 and Pd-L1 protein on AMs was increased after erlotinib therapy, which perhaps was an adaptive immune response to the inflammatory microenvironment caused by erlotinib (85). Interestingly, PD-L1 levels may even be affected by the concentration of EGFR-TKIs, in which a high concentration downregulates the expression of PD-L1, while a low concentration upregulates PD-L1 either in EGFR-MT cell lines or in animal tumors (104).

Further, an upward trend of PD-L1 was usually observed when NSCLC cells became resistant to TKIs (63, 105). Similar to a higher PD-L1 expression in gefitinib-resistant cells (106), PD-L1 expression markedly increased in patients with developed gefitinib resistance (63, 95). A retrospective clinical data also showed that six of 15 cases were found with increased PD-L1 expression after the failure of EGFR-TKI (61). Several different regulatory mechanisms of PD-L1 expression by resistant mechanisms of EGFR-TKIs have been proved. In acquired EGFR-TKI resistance induced by mechanisms of hepatocyte growth factor (HGF) and c-MET amplification, both the MAPK and PI3K pathways were involved in PD-L1 upregulation. In contrast, for EGFR-T790M (another EGFR-TKI resistance mechanism), the NF-kappa B pathway in addition to the above-mentioned two pathways was involved in the upregulation of PD-L1 (63). The PD-L1 expression in resistant NSCLC cells was still upregulated after continuous TKI treatment, which further significantly inhibited T-cell proliferation but slightly promoted apoptosis. Furthermore, the ERK1/2 pathway instead of STAT3 was verified to be associated with induced PD-L1 expression in resistant lung cancer (105). The expression of PD-L1 may even be varied with the resistance mechanisms after TKI treatment. For example, PD-L1 expression was higher for patients with a negative T790M resistance than for patients with a positive T790M resistance after TKI treatments (67).

As the same with EGFR-TKIs, other TKIs targeted by KRAS or MET may restrain the expression of PD-L1 as well after sensitive treatments (107, 108). After acquiring resistance to ALK inhibitor, PD-L1 expression in ALK-positive NSCLC cell lines and tumors predictably gained an increase in protein levels (including total and surface protein) and mRNA levels (109). Moreover, cancer cells in both the tumor nest and stroma showed a dramatic PD-L1 over-expression after ceritinib resistance.

#### 3.2.3 CD47

The expression of CD47 on the surface of pre-apoptotic cells was significantly downregulated by gefitinib in favor of engulfing cancer cells more efficiently by IFN-conditioned DCs. Not unexpectedly, the expression levels of CD47 did not change significantly in resistant NSCLC cells, which failed to promote tumor cell phagocytosis by DCs (71).

#### 3.2.4 ILT4

Both the protein and mRNA expression of ILT4 in sensitive cells were markedly decreased by gefitinib and osimertinib in a concentration-dependent way (45).

### 3.3 Cytokines and chemokines

Cytokines and chemokines tend to be influenced by EGFR-TKIs as well. With the use of RNA-seq analysis, the IFN target gene sets were also inducted by diverse TKIs such as EGFR-TKIs and even ALK-TKIs, which might trigger an inflammatory program mediated by the MAPK pathway (86). Moreover, the upregulation of type I IFN was triggered *via* a RIG-I-TBK1-IRF3 pathway in EGFR-MT cells (104). The levels of other pro-inflammatory cytokines (e.g., TNF-α, IFN-γ, and IL-2) produced by CD4+ and CD8+ T cells may also increase after erlotinib treatment (85). Pro-inflammatory cytokine IL-12p40 also had an increased secretion after erlotinib treatment (85). IL-6, secreted majorly by macrophages in the TME, is greatly involved in proliferation, apoptosis, invasion, angiogenesis, EMT, and metastasis *via* immunosuppression. Being a first-generation EGFR-TKI, icotinib could induce the secretion of IL-6, resulting in the activation of the STAT3 pathway through Src activation in EGFR-TKI-sensitive NSCLC cells (110). IL-8 or CXCL8 is involved in chemotaxis, DC migration, induction of phagocytosis, degranulation of neutrophils, and potentiation of acute inflammatory reactions. Both the secretion of IL-8 and the levels of IL-8 receptor alpha (CXCR1) were enhanced in the erlotinib-resistant NSCLC cells *via* upregulation of p38 MAPK signaling (111). Tumor necrosis factor (TNF) is a cytokine broadly expressed in lung cancer, which is secreted by malignant cells as well as other cells in the TME and may promote the growth of tumors. Whichever type the EGFR mutant was, TNF gained a rapid increase as a universal response to EGFR-TKIs, i.e., erlotinib and afatinib. Mechanistically, erlo tinib could induce NF-κB activation *via* TNF, which would be regulated by NF-κB in a feed-forward loop in turn (112).

In an analysis of both mRNA and protein expression, CXCL10, which can recruit CD8+ effector T cells, was upregulated after treatments by either erlotinib or osimertinib in EGFR-mutated LUAD samples. However, the result was the opposite for CCL22, which can recruit Tregs (85). Similarly, CCL2 and CCL5, chemoattractants for T cells, were upregulated after erlotinib treatment concurrent with downregulation for CCL3 and CXCL1 (85). The expression of CXCL2 in AMs was enhanced after erlotinib treatment (85). CXCR4 was discovered to be elevated after gefitinib treatment in PC9 harboring EGFR mutation, and EMT was consequently induced in a dose- and time-dependent manner (113). Even CCL2 in serum was significantly increased in both the EGFRL858R-driven model and EGFR19DEL/T790M-driven model after corresponding sensitive EGFR-TKI treatments (89). Moreover, chemokines may vary with the transcription factors. A consistent reduction of CCL22 and JUN after erlotinib treatment verified this (22).

## 4 Conclusions

Altogether, the components in the TME are complex and interactive. Compared with non-mutated NSCLC, the TME of EGFR-MT NSCLC generally tends to be more immunosuppressive, displaying a poor response to ICIs. Different oncogenic driver mutations may result in various TMEs; more CD8+ T cells were infiltrating the TME of KRAS-mutated and MET-mutated tumors than EGFR-MT, for example. The TME of oncogenic driver mutated NSCLC may also be altered during treatment with TKIs, especially the well-studied EGFR-MT. Generally, the TME upon responsive TKI treatments may be remodeled as a “hot tumor”, which was revealed by the following evidence: the immune-activated components including tumor-infiltrating immune cells, immunomodulatory molecules, cytokines, or chemokines in the TME were shown either with an increased number or with an upregulated function. In contrast, the immunosuppressive components decreased or appeared with impaired activity. Along with more comprehensive studies, the change of the TME after treatment with TKIs may even be dynamic and dependent on the treatment period, the sensitivity, or the regimen of TKIs. As a result, we should devote more attention to finding the most key component, which is involved in the regulation of the TME in oncogenic driver mutated NSCLC and the most suitable window for the administration of immune checkpoint inhibitors in our future investigations.

## Author contributions

DL, SC, JT, DS, and FL designed the study and wrote the protocol. SC and TY drafted the manuscript. DL, WL, and NY revised the manuscript content. All authors read and approved the final manuscript.
